# Numerical Analysis of Micro-Residual Stresses in a Carbon/Epoxy Polymer Matrix Composite during Curing Process

**DOI:** 10.3390/polym14132653

**Published:** 2022-06-29

**Authors:** Paulo Teixeira Gonçalves, Albertino Arteiro, Nuno Rocha, Luis Pina

**Affiliations:** 1 Institute of Science and Innovation in Mechanical and Industrial Engineering (INEGI), Rua Dr. Roberto Frias, 4200-465 Porto, Portugal; nrocha@inegi.up.pt (N.R.); luis.pina@strix.pt (L.P.); 2 Departamento de Engenharia Mecânica, Faculdade de Engenharia, Universidade do Porto, Rua Dr. Roberto Frias, 4200-465 Porto, Portugal

**Keywords:** curing simulation, polymer matrix composites (PMCs), micro-residual stresses, representative volume elements, computational micromechanics, constitutive modeling, CFRP

## Abstract

The manufacturing process in thermoset-based carbon fiber-reinforced polymers (CFRPs) usually requires a curing stage where the material is transformed from a gel state to a monolithic solid state. During the curing process, micro-residual stresses are developed in the material due to the different chemical–thermal–mechanical properties of the fiber and of the polymer, reducing the mechanical performance of the composite material compared to the nominal performance. In this study, computational micromechanics is used to analyze the micro-residual stresses development and to predict its influence on the mechanical performance of a pre-impregnated unidirectional CFRP made of T700-fibers and an aeronautical grade epoxy. The numerical model of a representative volume element (RVE) was developed in the commercial software Abaqus^®^ and user-subroutines are used to simulate the thermo-curing process coupled with the mechanical constitutive model. Experimental characterization of the bulk resin properties and curing behavior was made to setup the models. The higher micro-residual stresses occur at the thinner fiber gaps, acting as triggers to failure propagation during mechanical loading. These micro-residual stresses achieve peak values above the yield stress of the resin 55 MPa, but without achieving damage. These micro-residual stresses reduce the transverse strength by at least 10%, while the elastic properties remain almost unaffected. The numerical results of the effective properties show a good agreement with the macro-scale experimentally measured properties at coupon level, including transverse tensile, longitudinal shear and transverse shear moduli and strengths, and minor in-plane and transverse Poisson’s ratios. A sensitivity analysis was performed on the thermal expansion coefficient, chemical shrinkage, resin elastic modulus and cure temperature. All these parameters change the micro-residual stress levels and reduce the strength properties.

## 1. Introduction

Fiber-reinforced polymers (FRPs) are commonly used in aerospace and high-performance applications due to their higher strength-to-weight ratio. They are made with a fiber reinforcement embedded in a polymeric matrix. This reinforcement ranges from short fibers, continuous long fibers and fabrics. The most common fiber reinforcement materials are glass fibers (GFRPs), carbon fibers (CFRPs) and other organic fibers such as Kevlar, Dyneema, etc. [1]. Composite materials possess some advantages related to traditional (metallic) materials in addition to the weight reduction, including toughening for impact, fatigue resistance, corrosion resistance, electromagnetic transparency, erosion and wear resistance, acoustic and vibration damping, low thermal expansion, among others [1,2,3,4]. However, the most important advantage of FRPs is their tailoring ability to fit design requirements.

Two families of polymeric matrices are commonly used in FRPs, namely thermoplastics and thermosets. While thermoplastics are processed by melting the polymer, and cooling it down to its final configuration, thermoset-based composites are manufactured with a non-reversible thermo-curing process where the polymer is transformed from a gel to a monolithic material. The latter have been the preferred option in aerospace applications due to their easier processing characteristics.

Inadequate manufacturing processes in composite materials generate low-quality products and performance reduction in as-manufactured conditions due to residual stresses, voids formation, or incomplete curing. This performance reduction can lead to design failure because material properties were overestimated, or because the geometry does not fit the requirements [5,6]. Linking of composites manufacturing simulation with in-service performance is a recent research topic that has become affordable due to increased computing power, but we still lack a complete understanding of how it influences the final performance.

Focusing on carbon fiber-based thermoset composite systems (CFRPs), micro-residual stresses appear at the microstructural level due to the mismatch between the thermal expansion coefficients of the fiber and the polymeric matrix, and due to the chemical shrinkage, that takes place during the curing process [7,8,9,10,11]. Additional residual stresses can be obtained in composite laminates due to the anisotropy of the plies, but they are out of the scope of this work, which will focus on the micro- (constituents) scale of polymer matrix composites.

The curing process is inherently a thermo-chemical phenomenon for thermoset polymers where an exothermic chemical reaction starts at a given temperature and finishes when the polymerization process ends. One of the most commonly used approximation models for polymer curing was proposed by Kamal [12]. This model relates the curing state in the material with the time and temperature evolution. Currently, several works can be found in the literature regarding the experimental characterization of the curing behavior of polymeric resins, including epoxies [13,14,15,16,17,18,19,20]. Many of them use differential scanning calorimetry (DSC) and dynamical mechanical analysis (DMA) to identify the curing behavior and the evolution of the elastic modulus.

Experimental works to measure actual residual stresses at micro-scale level are not easy to perform due to the reduced length scale at which they occur. For instance, Minakushi [21] performs fiber-optic based tests to measure cure shrinkage in fiber-reinforced laminates, obtaining local strain measurements in the fiber direction and through the thickness, while Seers et al. [22] presents a recent review of the measurement techniques of residual stresses in composites, most of them relying on macro-measurements to obtain indirect measures of the micro-residual stresses. Similar difficulties arise regarding the characterization of the mechanical properties of the material at micro-scale level due to size effects [23], especially for the fracture toughness.

Numerical simulation of the properties evolution with curing is a current research field. To the best of the authors’ knowledge, Ersoy et al. [8] is among the first studies to deal with these simulations, considering only the elastic part of the material. Yuan et al. [24,25] uses viscoelastic constitutive modeling for the elastic modulus evolution during curing and representative volume elements (RVEs) consisting of one fiber or multiple fibers coupled with a multi-scale approach to capture temperature profiles from the macro-scale.

A similar approach was followed by Hui et al. [26], who also use a multi-scale approach to analyze the heat transfer effects of the curing process at the micro-scale level. They include an extended Drucker–Prager model to account for the polymer failure response. Danzi et al. [10] instead used a more advanced material constitutive model that accounts for the plastic response and failure of the polymer matrix, a model that was previously proposed by Melro et al. [27] and has been used by several authors to simulate the mechanical response of epoxies. All these works converge in the usage of RVE models [28,29] to analyze the mechanical response of the composite material at the micro-scale level with the appropriate boundary conditions (BCs) to guarantee solution consistency [30].

Another relevant topic relates to the methodologies used to estimate the effective macro-scale mechanical properties and the imposed BCs. Volumetric average measures of stress and strain fields are the most commonly used technique to extract the effective strain and stress response [28]. Second order homogenization theories are also available in the literature [31] but are not considered in this work because they require the usage of strain gradient measures, which falls outside the scope of this work.

Considering first order approximations, several authors have studied the influence of the BCs in the effective strain–stress response, concluding that the periodic boundary conditions (PBCs) give the best compromise between accuracy and computational simplicity [32,33]. However, the usage of PBCs has limitations in the simulation of strain localization phenomena such as damage propagation, because they over-constrain the strain field at the boundaries [28,32,34]. Nevertheless, PBCs provide accurate estimates up to failure initiation, which is the focus of this work.

Here, a micromechanical model is developed using the commercial software Abaqus^®^ and user-material (UMAT) subroutines to simulate the curing process and estimate the micro-residual stresses and its influence on the mechanical performance of an aerospace grade epoxy resin. A distinction between residual (macro-scale) and micro-residual (micro-scale) stresses is made because the micro-residual stresses are developed inside the RVE with traction-free boundary conditions.

Using the methodology proposed by Danzi et al. [10] that couples the polymer constitutive formulation proposed by Melro et al. [27] with the curing kinetics model, an enhanced temperature dependence of the material properties, especially for the elastic properties, is included in this work to appropriately model the shrinkage and the cooling processes.

The effective strain and stress measures are extracted directly from the PBCs output, i.e., the strain is obtained from the strain of the master node, and the stress from the force-conjugate measure of the applied strain, instead of using volumetric averages that are not appropriate when strain localization is achieved. The predictions obtained are then compared with experimental data obtained from standardized composite macro-scale tests.

A detailed analysis of the RVE response during the curing process is also performed using three different RVE geometries. Instantaneous measures of the effective macro-strain are extracted to see effective material expansion and contraction, besides the typical stress analysis. This analysis shows how the constituents are subjected to local stresses while keeping the macro effective stress equal to zero.

Additionally, due to the uncertainties of the material parameters at micro-scale level, a sensitivity analysis is performed in this work, to assess the influence of the thermal expansion coefficient, chemical shrinkage, resin elastic modulus and cure temperature on the residual stresses and the posterior effective mechanical properties, to complement the overall analysis of the micro-residual stresses.

The following section describes the materials, the constitutive modeling approach, the finite element procedures and the micro-residual stress analysis methodology. Then, the results are analyzed to highlight the micro-residual stresses distribution inside the RVE, and to link their influence on the effective mechanical properties. Finally, conclusions are drawn based on the discussion of the principal findings of this study.

## 2. Materials and Methods

### 2.1. Materials Selection

The fiber-reinforcement system selected for the experiments and simulations consists on the Toray T700 standard modulus carbon fiber. The parameters used in the current analysis are taken from the datasheet [35] and other previous works [10,28,36]. These parameters are summarized in Table 1, where $CTE_{L}$ and $CTE_{T}$ are, respectively, the longitudinal and transverse coefficients of thermal expansion, $E_{L}$ and $E_{T}$ are, respectively, the longitudinal and transverse elastic moduli, $v_{L}$ is the longitudinal Poisson’s ratio, $G_{L}$ and $G_{T}$ are the longitudinal and transverse shear moduli.

The polymer matrix used in this study corresponds to an aeronautic grade epoxy resin. Table 2 shows the material parameters used for model setup, where CTE is the coefficient of thermal expansion, $T_{g}$ is the glass transition temperature, $E_{m}$ is the elastic modulus, $v_{m}$ is the elastic Poisson’s ratio, $v_{p}$ is the plastic Poisson’s ratio, $S_{y+}$ and $S_{y-}$ are the tensile and compressive yield strengths and $S_{u+}$ and $S_{u-}$ are the tensile and compressive ultimate strengths, $\varepsilon_{f}$ is the failure strain at $S_{u+}$ and $G_{flm}$ is the fracture toughness. The mechanical properties for the pure resin were extracted from the datasheet, from dog-bone tensile tests and from the literature [10,27]. The properties reported in Table 2 correspond to the nominal properties of the matrix in as-manufactured conditions. (Degree of cure φ=1).

### 2.2. Constitutive Models

#### 2.2.1. Carbon Fibers

The fibers are modeled using a transversely isotropic elastic constitutive relation. This is a common approach for RVE modeling of composites whose transverse failure is controlled by the polymer matrix and its interface with the fibers. Equation (1) shows the elastic compliance matrix considering that direction 1 is the fiber direction. The Poisson’s ratio $v_{T}$ can be estimated from $G_{T}=E_{T}/\left(2\left(1+v_{T}\right)\right)$

(1) $$\left(\begin{array}{c} \varepsilon_{11}\\ \varepsilon_{22}\\ \varepsilon_{33}\\ \varepsilon_{12}\\ \varepsilon_{13}\\ \varepsilon_{23} \end{array}\right)=\left(\begin{array}{cccccc} 1/E_{L} & -v_{L}/E_{L} & -v_{L}/E_{L} & 0 & 0 & 0\\ -v_{L}/E_{L} & 1/E_{T} & -v_{T}/E_{T} & 0 & 0 & 0\\ -v_{L}/E_{L} & -v_{T}/E_{T} & 1/E_{T} & 0 & 0 & 0\\ 0 & 0 & 0 & 1/2/G_{L} & 0 & 0\\ 0 & 0 & 0 & 0 & 1/2/G_{L} & 0\\ 0 & 0 & 0 & 0 & 0 & 1/2/G_{T} \end{array}\right)\left(\begin{array}{c} \sigma_{11}\\ \sigma_{22}\\ \sigma_{33}\\ \sigma_{12}\\ \sigma_{13}\\ \sigma_{23} \end{array}\right)$$

#### 2.2.2. Polymer Matrix

The curing process in thermoset resins is usually an exothermic reaction process. It is normally characterized by the total curing reaction enthalpy $H_{tot}$, which is the total heat released by the curing reaction. If the instantaneous reaction enthalpy is defined as H(t,T), hence the curing process variable φ is defined as given in Equation (2). This parameter ranges from φ=0 when curing is not initiated, and φ=1 when the cured state is fully achieved.

(2) $$\varphi =\frac{H\left(t,T\right)}{H_{tot}}$$

The model proposed by Kamal [12] is used to describe the evolution of the curing state φ(t) with a similar approach to the one used by Danzi et al. [10]. The curing kinetics is given in Equation (3):

(3) $$\frac{d\varphi }{dt}=\left(k_{1}\left(T\right)+k_{2}\left(T\right)\varphi^{m}\right){\left(1-\varphi \right)}^{n}$$

The temperature dependent functions $k_{1}\left(T\right)$ and $k_{2}\left(T\right)$ are given in Equations (4) and (5). They can represent different reaction processes such as a cure for $k_{1}\left(T\right)$, and a post-cure for $k_{2}\left(T\right)$.

(4) $$k_{1}\left(T\right)=A_{1}\exp\left(-\frac{\Delta E_{1}}{R\cdot T\left(t\right)}\right)$$

(5) $$k_{2}\left(T\right)=A_{2}\exp\left(-\frac{\Delta E_{2}}{R\cdot T\left(t\right)}\right)$$

where:

$A_{1}$; $A_{2}$: Reaction velocities [1/s].
$\Delta E_{1}$; $\Delta E_{2}$: Activation energies [J/mol]
m; n: Fitting exponents
R: Universal gas constant [8.31432 J/mol.K]
T(t): Temperature

The parameters of the curing kinetics model are calibrated from differential scanning calorimetry tests.

On the other hand, it is also necessary to relate the mechanical properties evolution with the curing state. A phenomenological approach [37] is proposed to link the evolution of the mechanical properties with the curing state as given in Equation (6). The shift function S(φ) ranges from S(0)=0 when no curing is achieved and, S(1)=1 when the cure is completely achieved. This equation uses an arctan function because it approximates the actual experimental data shape and fulfills the boundary limit values (0 to 1).

(6) $$S\left(\varphi \right)=\frac{\arctan\left(\beta \left(\varphi -\gamma \right)\right)+\arctan\left(\beta \cdot \gamma \right)}{\arctan\left(\beta \cdot \gamma \right)+\arctan\left(\beta \left(1-\gamma \right)\right)}$$

The parameters β and γ can be obtained by fitting experimental data. β represents the slope and γ a phase shift to account for the delay of the property evolution with the actual curing state. The experimental data is obtained by DMA, but it can also be obtained by tensile tests at different curing states.

Therefore, the instantaneous elastic modulus E(φ) is obtained by Equation (7), the Poisson’s ratio by Equation (8) and the chemical shrinkage by Equation (9).

(7) $$E\left(\varphi \right)=E_{m}\cdot S\left(\varphi \right)$$

(8) $$v\left(\varphi \right)=S\left(\varphi \right)\;v_{c}+\left(1-S\left(\varphi \right)\right)v_{0}$$

(9) $$\alpha \left(\varphi \right)=\alpha_{m}\cdot S\left(\varphi \right)$$

where:

v(0)=0.5 Poisson’s ratio at un-cured state
$v\left(1\right)=v_{m}$ Poisson’s ratio at cured state
E(0)=0 Elastic modulus at un-cured state
$E\left(1\right)=E_{m}$ Elastic modulus at cured state
α(0)=0 Chemical shrinkage at un-cured state
$\alpha \left(1\right)=\alpha_{m}$ Chemical shrinkage at cured state.

The properties at completely cured state $v_{m}$, $E_{m}$, $\alpha_{m}$ are the nominal properties of the cured material at a given temperature. To avoid numerical issues, it is considered that $E\left(0\right)=10^{-3}\cdot E_{m}$.

The initial elastic behavior is defined by a linear isotropic relation between the stress tensor, σ, and the elastic strain, $\varepsilon_{e}$ as given in Equation (10):

(10) $$\sigma =\left[D_{e}\right]\cdot \varepsilon_{e}$$

where $D_{e}$ is the isotropic elastic tensor typically defined by the Young’s modulus E, and the Poisson’s ratio v.

A paraboloidal yield criterion Φ, Equation (11), is used to account for hydrostatic pressure effects:

(11) $$\Phi \left(\sigma ,\sigma_{C},\sigma_{T}\right)=6\cdot J_{2}+2\cdot I_{1}\cdot \left(S_{y-}-S_{y+}\right)-2\cdot S_{y-}\cdot S_{y+}=0$$

where $S_{y-}$ and $S_{y+}$ are the compressive and tensile yield strengths of the material, respectively, $J_{2}$ is the second invariant of the deviatoric stress tensor, and $I_{1}$ is the first invariant of the stress tensor.

The flow rule ψ is given in Equation (12). It is non-associative to account for the volumetric deformation:

(12) $$\psi \left(\sigma \right)=3\cdot J_{2}+\alpha /9\cdot {\left(I_{1}\right)}^{2}$$

where α corresponds to the material parameter that adjusts the volumetric component of the plastic flow. It is dependent of the plastic Poisson’s ratio, and it is given by:

(13) $$\alpha =\frac{9}{2}\left(\frac{1-2v_{p}}{1+v_{p}}\right)$$

The yield strengths are used to define the yield surface. Hardening is considered to affect both yield strengths and the increment of equivalent plastic strain is:

(14) $$\Delta {\bar{\varepsilon }}_{p}=\sqrt{k\Delta \varepsilon_{p}:\Delta \varepsilon_{p}}$$

where k ensures that the equivalent plastic strain is the same to that obtained in the uniaxial test:

(15) $$k=\frac{1}{1+2v_{p}^{2}}$$

The yield stress hardening curve is approximated with a potential law as:

(16) $$S_{y-/+}=Sy_{0}+K_{s}\cdot {\left({\bar{\varepsilon }}_{p}\right)}^{n_{c}}$$

where $Sy_{0}$ is the initial yielding stress, $K_{s}$ is the hardening coefficient and $n_{c}$ is the hardening exponent. One curve is used for the tensile part, and another curve is used for the compressive case.

The damage evolution model proposed by Melro et al. [27] enforces the damage to develop only in the material elastic modulus instead of the complete elastic tensor, an approach commonly followed by other continuum damage mechanics models usually implemented in a finite element method context [38,39].

The thermodynamic free energy potential $D_{m}$ is given in Equation (17):

(17) $$D_{m}=\frac{\sigma_{11}^{2}+\sigma_{22}^{2}+\sigma_{33}^{2}}{2E_{m}\left(1-d_{m}\right)}-\frac{v_{m}}{E_{m}}\left(\sigma_{11}\sigma_{22}+\sigma_{22}\sigma_{33}+\sigma_{33}\sigma_{11}\right)+\frac{1+v_{m}}{E_{m}\left(1-d_{m}\right)}\left(\sigma_{12}^{2}+\sigma_{13}^{2}+\sigma_{23}^{2}\right)+D_{m}^{P}$$

where σ corresponds to the stress tensor, $E_{m}$ and $v_{m}$ correspond to the undamaged material elastic modulus and Poisson’s ratio, respectively, and $d_{m}$ corresponds to the damage variable. It is assumed that the damage process is de-coupled from the plastic dissipation process. Therefore, the plastic dissipation $D_{m}^{P}$ is not considered in the subsequent damage formulation.

The damage initiation criterion $\Phi_{m}^{d}$ is given by Equation (18). This equation has a similar shape compared to the yield criterion, with the difference that $S_{u-}$ and $S_{u+}$ correspond to compressive and tensile ultimate strengths, while $\tilde{I_{1}}$ and $\tilde{J_{2}}$ correspond to the effective un-damaged effective stress invariants in the material.

(18) $$\Phi_{m}^{d}=\frac{3\tilde{J_{2}}}{S_{u-}\cdot S_{u+}}+\frac{\tilde{I_{1}}\cdot \left(S_{u-}-S_{u+}\right)}{S_{u-}\cdot S_{u+}}$$

The damage evolution condition is given by Equation (19). The damage parameter $r_{m}$ ranges from 1, to satisfy the condition $F_{m}^{d}<0$ when no damage is present, to +∞ when the material is completely damaged.

(19) $$F_{m}^{d}=\Phi_{m}^{d}-r_{m}\leq 0$$

An exponential damage law is proposed to relate the damage parameter $r_{m}$ in the range [1,+∞] with the damage variable $d_{m}$ in the range [0,1]. Equation (20) presents the exponential law where $A_{m}$ is a calibration parameter to regularize the volumetric damage dissipated energy in the element with the fracture energy.

(20) $$d_{m}=1-\frac{\exp\left(A_{m}\left(3-\sqrt{7+2r_{m}^{2}}\right)\right)}{\sqrt{7+2r_{m}^{2}}-2}$$

The damage dissipated energy $Y_{m}$ can be obtained differentiating Equation (17), as shown in Equation (21).

(21) $$Y_{m}=\frac{\partial D_{m}}{\partial d_{m}}=\frac{\sigma_{11}^{2}+\sigma_{22}^{2}+\sigma_{33}^{2}}{2E_{m}{\left(1-d_{m}\right)}^{2}}+\frac{1+v_{m}}{E_{m}{\left(1-d_{m}\right)}^{2}}\left(\sigma_{12}^{2}+\sigma_{13}^{2}+\sigma_{23}^{2}\right)$$

The total dissipated energy due to the fracture process $W_{f_{m}}$ can be calculated integrating the dissipated energy over the internal damage parameter as given in Equation (22). The total dissipated energy per unit volume should be equal to the surface fracture energy $G_{flm}$ divided by the characteristic element size $l^{e}$ to ensure a mesh-independent damage law.

(22) $$W_{f_{m}}=\int_{1}^{+\infty }Y_{m}\cdot \frac{\partial d_{m}}{\partial r_{m}}dr_{m}=\frac{G_{flm}}{l^{e}}$$

#### 2.2.3. Thermo-Curing Coupling Strategy

The total strain mechanical strain $\varepsilon_{t}$ cam be decomposed as shown in Equation (23) where $\varepsilon_{e}$ is the elastic strain, $\varepsilon_{p}$ is the plastic strain, $\varepsilon_{th}$ is thermal expansion induced strains and $\varepsilon_{sh}$ are the chemical contraction-induced strains.

(23) $$\varepsilon_{t}=\varepsilon_{e}+\varepsilon_{p}+\varepsilon_{th}+\varepsilon_{sh}$$

To couple the curing phenomena in the material with the mechanical response, the elastic constitutive tensor $D_{e}\left(\varphi ,T\right)$ is assumed to be a function of the curing level and the temperature as shown in Equation (24), where S(φ) is the shift function and β(T) is the temperature dependence function of the elastic modulus.

(24) $$D_{e}\left(\varphi ,T\right)=D_{e}^{0}\cdot S\left(\varphi \right)\cdot \beta \left(T\right)$$

The temperature dependence function β(T) is given in Equation (25) and it is a function of the coefficient $\beta_{E}$ for a given reference temperature $T^{*}$. It was, originally proposed by Bai et al. [37]. $\beta_{E}$ is extracted by fitting the variation of the storage modulus with temperature obtained from a DMA test on a sample of cured resin. The obtained value for $\beta_{E}=1.98$.

(25) $$\beta \left(T\right)=\left(1+\beta_{E}\cdot \log\left(\frac{T}{T^{*}}\right)\right)$$

Due to the nature of the curing problem, the equilibrium stress rate should be written in rate form similar to an hypoelastic law $\dot{\sigma }\left(\dot{\varepsilon },\dot{T}\right)$, because the elastic constitutive tensor changes with time and temperature [10,40]. With this approach, material hardening due to curing and softening due to a temperature increase is appropriately considered, even at constant mechanical strain.

(26) $$\dot{\sigma }=\frac{\partial \sigma }{\partial \varepsilon }\cdot \dot{\varepsilon }+\frac{\partial \sigma }{\partial T}\cdot \dot{T}$$

The instantaneous stress is approximated by $\sigma =D_{e}\cdot \varepsilon_{e}$, to be differentiated with temperature. In this procedure, the elastic strain is assumed to be constant during temperature variation $\partial \varepsilon_{e}/\partial T=0$. For $\dot{T}<0$, $\partial D_{e}/\partial T>0$ which implies a stress increase for fixed elastic strain. This phenomenon is unrealistic, hence, for $\dot{T}<0$, $\partial D_{e}/\partial T=0$:

(27) $$\frac{\partial \sigma }{\partial T}\cdot \dot{T}=\left(\frac{\partial D_{e}}{\partial T}\cdot \varepsilon_{e}+D_{e}\cdot \frac{\partial \varepsilon_{e}}{\partial T}\right)\cdot \dot{T}=\left(\frac{\partial D_{e}}{\partial T}\cdot \varepsilon_{e}\right)\cdot \dot{T}$$

Finally, during the curing process the elastic strain measure cannot be directly related with the initial/undeformed configuration. Hence, an instantaneous elastic strain measure can be calculated using the compliance tensor (inverse of elastic tensor) for the current stress state. With this approach the undeformed configuration is kept as the stress-free configuration which is not the initial configuration. This formulation is compatible with the plasticity theory but without damage. At damage onset, plasticity and curing are not allowed to develop [6].

(28) $$\varepsilon_{e}={\left(D_{e}\right)}^{-1}\cdot \sigma$$

The stress increment is calculated using a trapezoidal integration scheme as shown in Equation (29). $\langle \Delta T^{n+1}\rangle$ means only positive changes of temperature.

(29) $$\sigma^{n+1}=\sigma^{n}+\frac{1}{2}\cdot \left(D_{e}^{n}+D_{e}^{n+1}\right)\cdot \Delta \varepsilon^{n+1}+\left(\frac{\partial D_{e}}{\partial T}\cdot \varepsilon_{e}\right)\cdot \langle \Delta T^{n+1}\rangle$$

### 2.3. Finite Element Model

**Figure 1:**
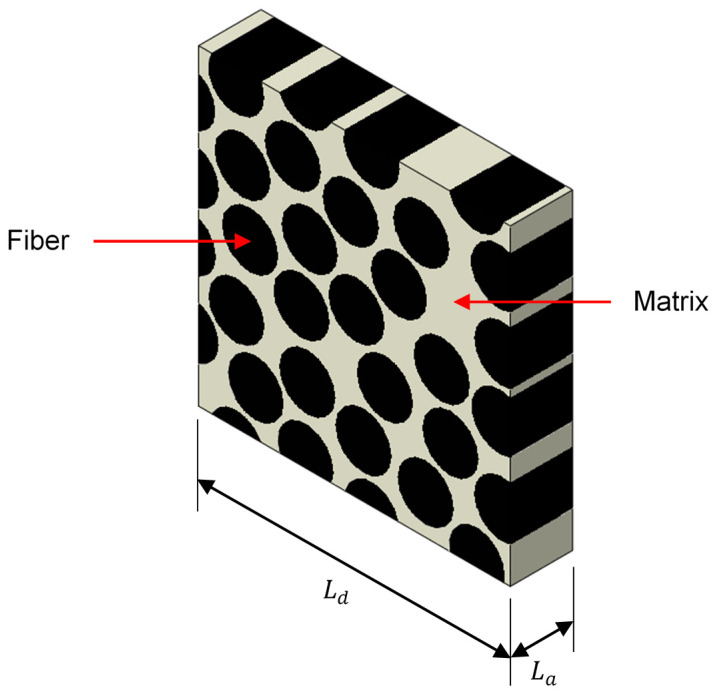
Example of a RVE.

The RVE geometry is defined using a constant fiber diameter $d_{f}$ and a square transverse section. Setting the number of fibers $n_{f}={\bar{n}}_{f}^{2}$ (to achieve a square RVE) and fiber volume fraction Vf, the side size $L_{d}$ of the RVE can be explicitly calculated with Equation (30). Next, to generate the random distribution of fibers inside the RVE, the methodology proposed by Calatanotti [29] is implemented. Figure 1 shows an example of a typical RVE with $n_{f}=25$ and Vf=60%, where $L_{a}$ corresponds to the length of the RVE in the fiber direction.

(30) $$L_{d}=\sqrt{\frac{n_{f}\cdot \pi \cdot d_{f}^{2}}{4\cdot Vf}}$$

Additional constraints for the RVE generation include a maximum fiber separation of $4\times d_{f}$ to avoid unrealistic resin-rich regions and a minimum fiber separation of $1.1\times d_{f}$ to avoid meshing difficulties. Lower fiber separations can also be achieved if required, but considerations regarding a good discretization of the inter-fiber regions led to the adoption of this value.

PBCs are implemented to guarantee continuity in the displacement field locally without over-constraining the boundary faces. The PBCs are derived from the strain-displacement definition, applied to opposite faces as given in Equation (31).

(31) $$\begin{array}{c} \varepsilon_{ij}=\frac{du_{i}}{dx_{j}}=\frac{u_{i}^{A}-u_{i}^{B}}{L_{j}} \end{array}$$

The numerical implementation follows the procedure proposed by Barbero [30], where the nodal constrain equations are generated for each set of opposite faces, considering that edges and corners must be joined to guarantee the equations consistency. The general form of the constrain equations are given in Equation (32), where ${\left\{u_{i}\right\}}^{+f}$ corresponds to the displacement in the direction i at face f, while ${\left\{u_{i}\right\}}^{-f}$ corresponds to the displacement in the same direction and opposite face. $L_{f}$ is the length of the RVE in the direction f and $\varepsilon_{if}$ is the imposed strain component. The loading in the RVE is imposed via a master node.

(32) $${\left\{u_{i}\right\}}^{-f}-{\left\{u_{i}\right\}}^{+f}-L_{f}\cdot \varepsilon_{if}=0$$

The effective composite properties, incl. elastic tensor components, yield strength and ultimate strength, are identified from the force-displacement response. The material parameters are calculated by enforcing that the load-displacement behavior of an equivalent monolithic RVE matches the full model RVE. Instead of volume average strain or stress measures, the effective strain corresponds to $\varepsilon_{if}$ from Equation (32), and the force conjugate is obtained from the force reaction at the master node. The effective stress is obtained by dividing this force reaction by the associated cross-section area of the RVE.

Table 3 shows the test loading cases required to identify the selected material parameters during the current study. The effective elastic tensor is approximated with a transversely isotropic tensor, that is defined by five elastic constants, namely $E_{L}$, $E_{T}$, $v_{L}$, $G_{L}$ and $G_{T}$. Additionally, yielding stresses and failure stresses were also obtained for this loading conditions to show the influence of the manufacturing parameters on the effective inelastic deformation and strength properties of the composite. In the current study, attention will be given to the transverse properties, which are dominated by the matrix response. Hence, the results for the longitudinal modulus $E_{L}$ will not be included in the analysis.

**Figure 2:**
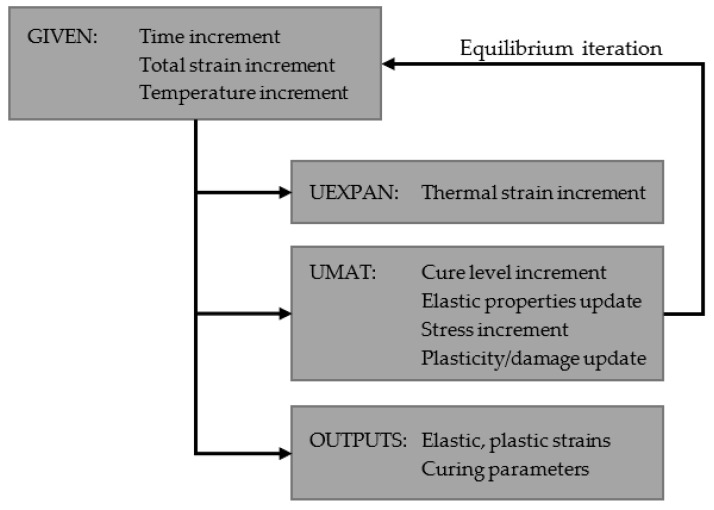
Model solution procedure.

The solution procedure to analyze the cure residual stresses begins with a thermomechanical analysis where the temperature curing cycle is imposed in the RVE domain. A pure mechanical analysis is considered because the temperature gradients inside the RVE are negligible. During this analysis step, the curing is simulated to determine the elastic properties evolution. The micro-residual internal stresses develop as a consequence of the interaction between the resin and the fibers. The PBCs are imposed in this step with a traction-free condition to allow the RVE to expand and shrink freely but conserving the periodicity. The constitutive model is completely implemented in a UMAT user subroutine, the shrinkage and thermal expansion via a UEXPAN user subroutine and the PBCs are implemented using a Python script to generate the input file. Figure 2 shows a flowchart of the solution procedure applied at each element integration point. The characteristic element size is given directly by the Abaqus solver in the UMAT subroutine, and it corresponds to the cubic root of the element volume.

After the curing step is completed, another analysis step is made to perform the test loading cases given in Table 3. The PBCs are kept the same but in this step a displacement-controlled load is applied in accordance, keeping the remaining degrees of freedom traction-free. To improve the computational efficiency, a restart point technique is used at the end of the curing step, to be used as the starting point of each loading case.

### 2.4. Micro-Residual Stress Analysis

The nominal curing cycle recommended by the material supplier, given in Table 4, is used to manufacture the coupons and to perform the micromechanical simulations.

After a preliminary RVE size analysis considering RVEs with 9, 16, 25 and 36 fibers, an RVE with $n_{f}=16$ gives the best compromise between computational cost and convergence of the results convergence. This definition is consistent with previous computational micro-mechanics works [10,28], leading to an RVE size $L_{d}\approx 4.58\cdot d_{f}$. The RVE thickness was defined to guarantee at least 3 finite elements along this direction, i.e., $L_{a}\approx 0.15\cdot d_{f}$, because the RVE thickness has a minor effect on the transverse properties [41], the ones of interest in this study.

**Figure 3:**
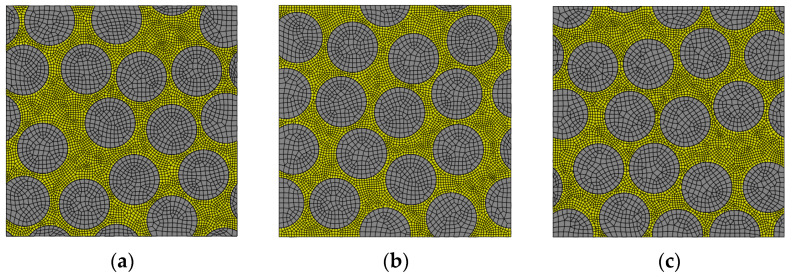
Randomly generated RVE geometries used for the micro-residual stress analysis. (**a**) RVE1, (**b**) RVE2 and (**c**) RVE3.

A set of three different statistically representative RVE geometries are used to perform all simulations, reducing the geometry dependence effects. Figure 3 shows the selected RVE geometries, namely RVE1, RVE2 and RVE3. After a mesh convergence analysis, the element size was set to be approximately 1/20 the fiber diameter for the matrix, and 1/10 for the fibers (to reduce computational costs). The average element size in the matrix is 0.00035 mm, which is defined in a compromise with the minimum fiber separation $1.1d_{f}$ to ensure at least two elements in the smaller gaps and avoid spurious artificial stresses due to badly shaped elements. Linear solid elements C3D8 from the Abaqus library were used. The matrix and the fiber are connected by tie constraints.

The fact that interface failure is not considered in these analyses can lead to over-estimations in the effective strength prediction of the material, because the interface strength is typically lower than the bulk resin strength [28,42]. Although the current modelling approach does not impede the consideration of interface failure, e.g., through cohesive interfaces, since no information regarding the interface properties for the current material system is available, and the evolution of the interface properties with curing state is, to the best of the authors’ knowledge, unknown, it was decided to focus the analysis on the evaluation of the micro-residual stresses developed during the curing process and their effects on matrix failure only.

A sensitivity analysis was performed to analyze the influence of the chemical shrinkage, thermal expansion coefficient, elastic modulus and cure temperature in the micro-residual stress state and mechanical properties. Table 5 shows the parameters used in this sensitivity analysis. Each parameter is arbitrarily ranged around the nominal value of the resin system.

### 2.5. Experimental Test Procedures

In order to calibrate the curing kinetics model, differential scanning calorimetry (DSC) tests were performed to un-cured resin samples following ASTM D3418. Next, to measure the instantaneous evolution of the storage modulus with curing, three DMA tests to un-cured resin samples are made, keeping the curing temperature fixed at 120 °C.

Additionally, coupon level tests were performed to measure the macro-mechanical properties of the corresponding composite system. The following tests were performed:

- Transverse tensile tests following ASTM D3039.
- Longitudinal shear tests following ASTM D3518.
- Transverse shear tests following ASTM D5379.

The coupons were extracted from composite plates manufactured following the curing cycle presented in Table 4 and trimmed by CNC machining.

## 3. Results and Discussion

### 3.1. Experimental Results

The curing model parameters for the epoxy resin used in this study were obtained by fitting the experimental data of the curing process measured with DSC tests. The parameters are given in Table 6.

**Figure 4:**
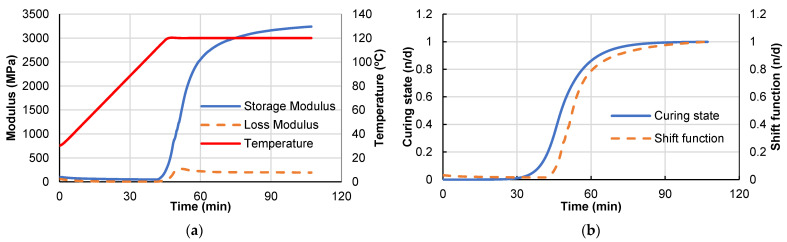
Results from DMA analysis. (**a**) Storage and loss modulus (**b**) Curing state and shift function.

Figure 4a shows the results of one representative DMA curing test. It shows how the elastic modulus begins to grow after the temperature of 100 °C is reached while the curing level develops.

Using the maximum obtained storage modulus and Equation (7) to obtain the instantaneous values of the shift function S(φ), Figure 4b shows the evolution of the shift function S(φ) and the curing state of the material with time. It shows the storage modulus follows the curing state response with some delay.

**Figure 5:**
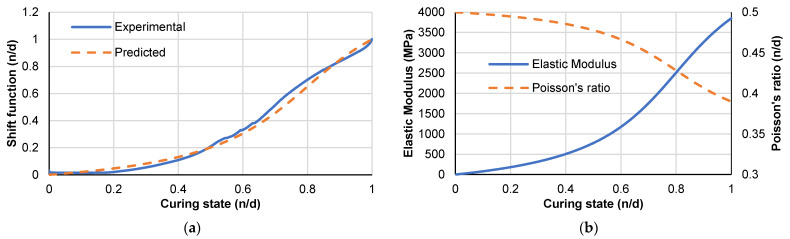
(**a**) Shift function for storage modulus and (**b**) Elastic properties evolution with curing level.

Accordingly, the shift function given in Equation (7) is fitted, obtaining the parameters *β* = 4 and *γ* = 0.8. A comparison between the experimental relation and the relation from Equation (7) is given in Figure 5a. Figure 5b shows the estimated mechanical properties of the resin system using Equations (7)–(9), which will be implemented in the numerical material models.

**Figure 6:**
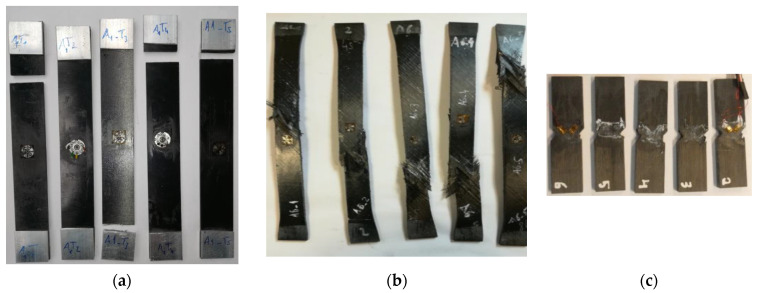
Coupons after testing. (**a**) Transverse tensile tests, (**b**) Longitudinal shear tests and (**c**) Transverse shear tests.

The tests coupons after mechanical testing are shown in Figure 6. Two transverse tensile coupons failed near the tabs; however, the measured strength has a low deviation, and the values are similar to the ones reported by the manufacturer and other test campaigns, making them suitable for the scope of this work. The failure modes for the longitudinal and transverse shear tests are within the expected behavior.

A summary of the experimental results is given in Table 7.

### 3.2. Residual Stress Analysis

During the curing process, the elastic modulus is developed at the same time the material expands due to the effects of the thermal expansion, and contracts due to chemical shrinkage. If the matrix material is completely free to deform it is not possible to generate stresses at the micro-mechanical level. However, in the case of fiber-reinforced composites, the fibers introduce local restrictions to the free deformation of the resin leading to the generation of local micro-residual stresses, the first stress generating mechanism. The second stress generating mechanism is the CTE incompatibility between the two constituents that becomes more relevant during the cooling-down stage.

**Figure 7:**
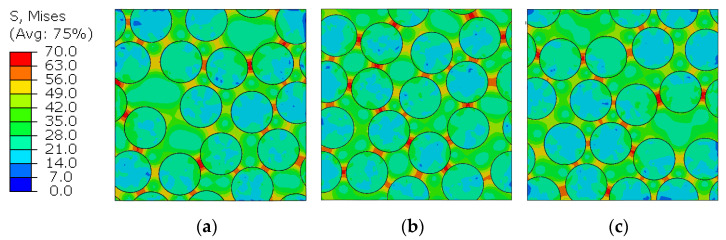
RVE micro-residual von Mises stress distribution for (**a**) RVE1, (**b**) RVE2 and (**c**) RVE3.

**Figure 8:**
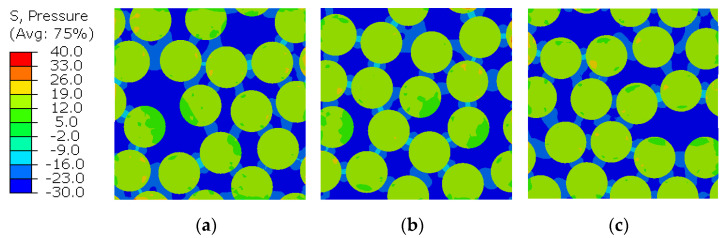
RVE micro-residual hydrostatic stress distribution for (**a**) RVE1, (**b**) RVE2 and (**c**) RVE3.

To analyze the generated micro-residual stresses, Figure 7 and Figure 8 show the stress distribution for the three different RVEs by means of the von Mises stress and the pressure stress. These measures were selected because they correspond the stress invariants that define the yielding and the failure criteria given in Equations (11) and (18).

The regions between the fibers concentrate higher deviatoric (von Mises) stresses in the matrix, being the most favorable regions to damage initiation and propagation. On the other hand, the hydrostatic stress distribution in the matrix is predominantly negative, which means that the bulk is in tensile state (remembering that $\sigma_{Pressure}=-1/3\;\mathrm{trace}\left(\sigma \right)$), while the fibers remain in compression, as expected since the whole RVE is in global equilibrium and under traction-free boundary conditions.

**Figure 9:**
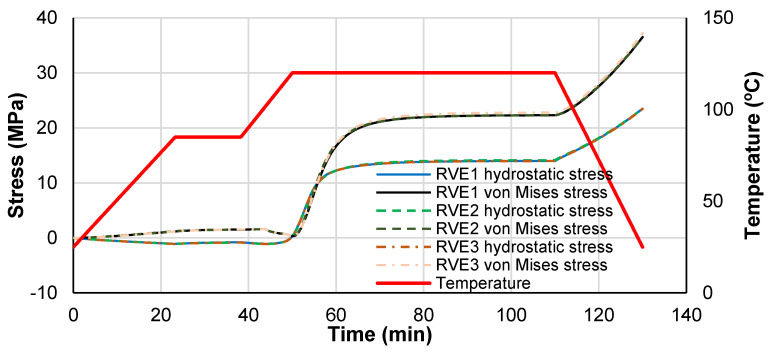
RVE average hydrostatic and von Mises stress evolution during the curing process.

**Figure 10:**
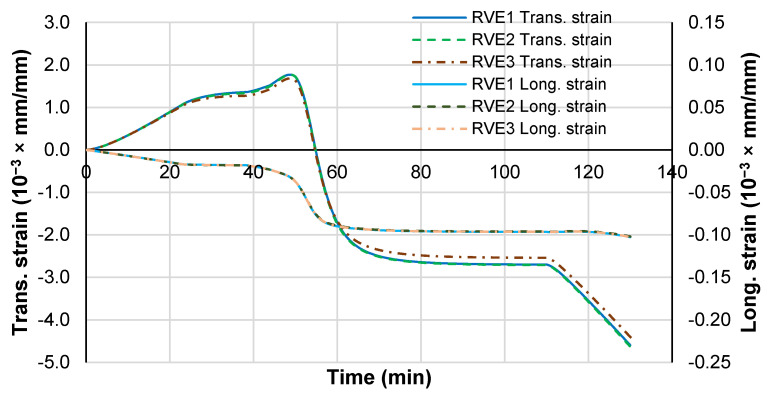
Effective RVE transversal and longitudinal strain evolution during curing process.

To investigate the stress components evolution during the curing process, Figure 9 shows the von Mises stress and the hydrostatic stress components evolution as a function of time, and Figure 10 shows the effective RVE strain measured at the master nodes. Temperature is also superposed to ease the analysis. In this analysis, the stress measures correspond to a volumetric average over the matrix volume region. During the heating stage prior to the initiation of the curing in the polymer, no stress is generated and the RVE expands due to the combined effect of the positive CTE of the fibers and resin in the transverse direction, while it contracts in the longitudinal direction due to the negative CTE of the fibers.

Next, the curing process begins, and the matrix shrinks at a constant temperature (inside the plateau), until the curing process stops, achieving a stress equilibrium state with a smaller volume. A cooling stage follows, where additional straining is retrieved, developing more micro-residual stresses.

The strain measure evolution with time and the degree of cure present trends similar to the strain measured experimentally with optical fibers by Minakushi [21].

Therefore, the matrix volumetric shrinkage is the precursor of the stress development during curing because it occurs at constant temperature, while the thermal contraction (CTE) is the stress precursor during cooling. The longitudinal strain deformation of the RVE is very low because it is dominated by the high stiffness of the fibers and their low negative CTE.

**Figure 11:**
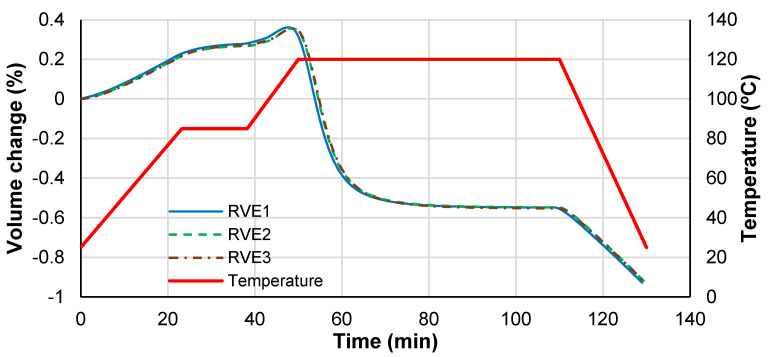
RVE volume change during curing process.

The chemical shrinkage for this resin is 2%, while the thermal contraction during cooling is only 0.6% (CTE multiplied by the temperature amplitude—from curing temperature to room temperature). However, the final volume reduction is around 0.9% estimated from the final volume of the RVE as presented Figure 11.

If a full restrained analysis is performed, the volume contraction is enough to cause material failure, because the failure strain is lower than 0.7%. For the current example, no damage is retrieved from the numerical results, and only small plastic straining is found in the stress concentration zones.

**Figure 12:**
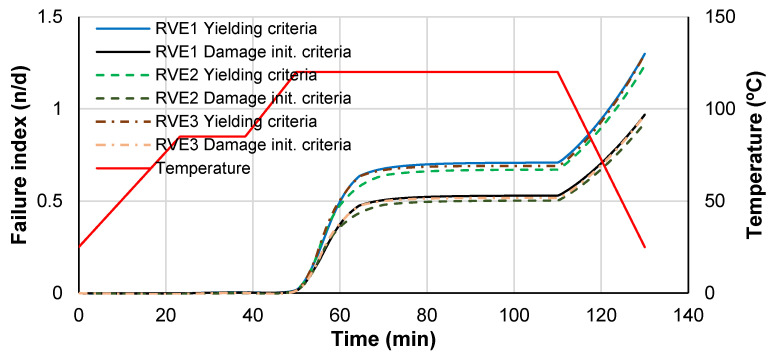
Failure indexes for yielding and damage initiation.

Other results that can be extracted from the curing analysis are the failure index measurements in the RVE given by Equations (11) and (18). For this purpose, Figure 12 shows the maximum failure index retrieved from the RVE as a function of time. It is clear from the previous figures that the micro-residual stresses achieve representative values especially between the narrowest fiber gaps and these stresses lead to the development of plastic strains. For the current example, no damage is achieved but the failure index is above 0.9.

### 3.3. Effective Mechanical Properties

To study the influence of the micro-residual stresses in the mechanical performance of the composite material, several simulations were performed using the selected RVEs subjected to different loading conditions as planned in Table 3, giving special attention to the properties dominated by the matrix behavior. Two sets of simulations are analyzed: the first corresponds to an analysis with the nominal material properties without considering the curing analysis and without the micro-residual stress effects, which is named “no-cure”, and another set that is analyzed using the micro-residual stresses and the actual material condition coming from the curing step analysis, named “cure”.

**Figure 13:**
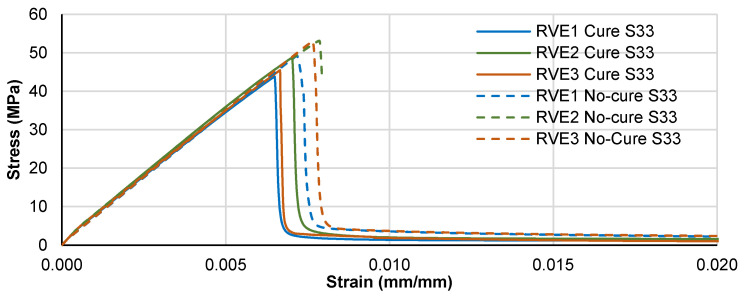
Transverse tensile stress-strain response comparison.

The stress–strain response for the transverse tensile loading case is presented in Figure 13. A direct comparison between the three different RVEs for the “no-cure” and “cure” condition is made. First, the overall response of the RVE is linear until failure, although the matrix resin is subjected to some plastic deformation. The elastic modulus remains unaffected because the material is completely cured, but the transverse tensile strength is reduced by the micro-residual stresses as expected.

**Figure 14:**
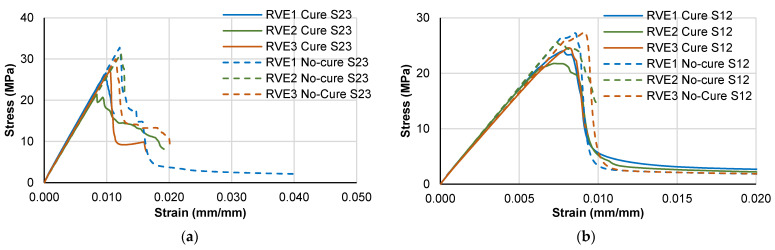
Shear stress-strain response comparison. (**a**) Transverse direction and (**b**) Longitudinal direction.

A similar response is retrieved for the shear loading simulations in both transverse and longitudinal directions, as shown in Figure 14. The shear modulus is practically unaffected by the micro-residual stresses and only the shear strength is reduced.

**Figure 15:**
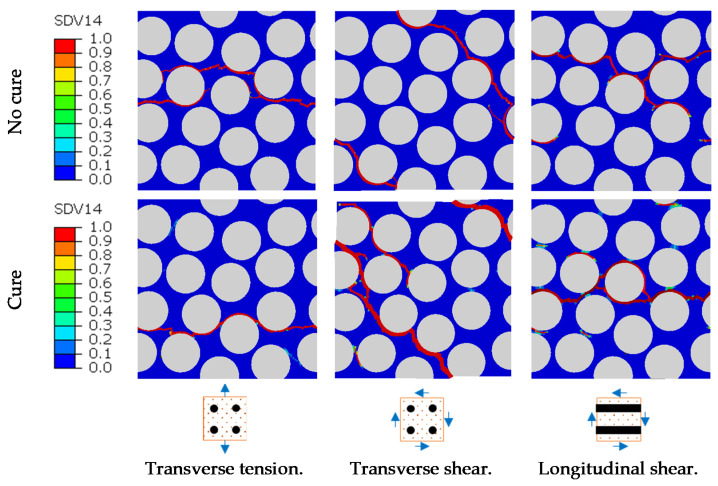
Damage patterns at failure for the different loading cases (SDV14 refers to the damage state variable).

Figure 15 shows the fracture pattern for each loading case for the “no-cure” and “cure” tests. A similar failure pattern is retrieved for both cases because it is given by the loading condition, with the only difference in the initiation point that changes the position of the crack bands. In the case of the cure analysis, the initiation point is influenced by the micro-residual stress levels, unlike the no-cure analysis.

To facilitate the comparison of results between both analysis sets, Table 8 shows a summary of the predicted effective mechanical properties and the experimental results. From the tensile tests, the transverse elastic modulus $E_{2}$ and the Poisson’s ratio $v_{21}$ from the experimental measurements are similar to the numerical predictions, the transverse tensile strength $S_{+UT}$ presents a reduction of 11% from the “no-cure” condition although the experimental measured value is slightly lower. This difference can be attributed to the effect of the fiber-matrix interface and other manufacturing defects which are not considered in the current analysis [28,42]. Another relevant result that should be highlighted is the fact that the tensile strength of the composite is almost 30% lower than the bulk matrix strength, a tendency that is commonly highlighted in the literature [9,10].

A similar trend is observed for the predicted transverse and longitudinal shear moduli, which are similar to the experimental results, while the strength measures present a reduction of 22% and 12%, respectively, when compared with the “no-cure” condition. Similarly, the experimental measured values are lower than the numerical predictions. The effective properties estimated considering the cure condition are closer to the experimental results.

These results show evidence that the micro-residual stresses in the RVE after the curing process can reduce the material strength and should be considered when performing homogenization procedures. Regarding the fracture response, further work is required to understand the damage mechanisms and the appropriate size effects that must be considered, because the application of the material models and mechanical properties from the macro-scale experimental measurements are straightforward for the elastic and strength parameters, but not for the fracture properties.

### 3.4. Sensitivity Analysis Results

A sensitivity analysis of the relevant material and process parameters that influence the curing micro-residual stresses is presented in this section. After a preliminary study following previous literature results [6], the material parameters that have a direct influence in the residual stresses are the CTE, the chemical shrinkage and the matrix elastic modulus. On the other hand, from the process point of view, since thermal gradients and transient effects are negligible at the RVE scale, the only relevant parameter is the cure temperature because it has a direct influence in the final curing level.

**Figure 16:**
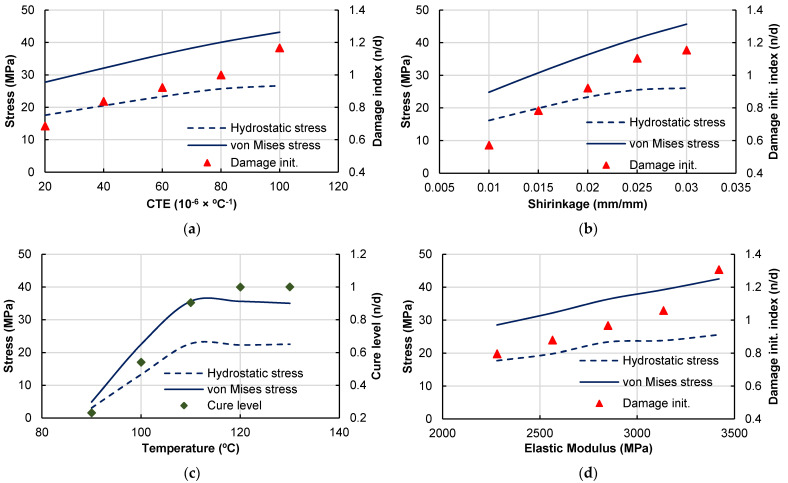
Micro-residual stresses and failure index/cure level sensitivity to material and process parameters. (**a**) Thermal expansion sensitivity results, (**b**) Chemical shrinkage sensitivity results, (**c**) Cure temperature sensitivity results, (**d**) Matrix elastic modulus sensitivity results.

Figure 16 shows the results for the sensitivity analyses for each of the selected variables regarding the micro-residual stress level and the failure index. The failure index corresponds to the maximum value of the failure initiation criteria, given by Equation (18), over the matrix region. The results presented for the stress measure corresponds to the volumetric average over the entire matrix region of the RVE.

A clear tendency to increase the micro-residual stresses arises from increasing the CTE and the chemical shrinkage. For higher levels of micro-residual stresses, damage is achieved, with the failure index becoming higher than unity.

The elastic modulus also has a relevant influence on the micro-residual stresses. The tendency shows that micro-residual stresses increase with increasing elastic modulus, including scenarios of damage onset. It is worth to note that the strength properties are kept constant during the sensitivity analyses.

The cure temperature has a different influence on the micro-residual stresses because it changes the degree of cure evolution and the final curing level in the matrix and not the mechanical response directly. This difference in the curing level changes the final elastic modulus, Poisson’s ratio and overall mechanical behavior of the material. For lower temperatures, keeping the cure cycle time unchanged, the cure level is reduced to 20%, a very low value, but (as known) the final properties would not be suitable for a composite material.

**Figure 17:**
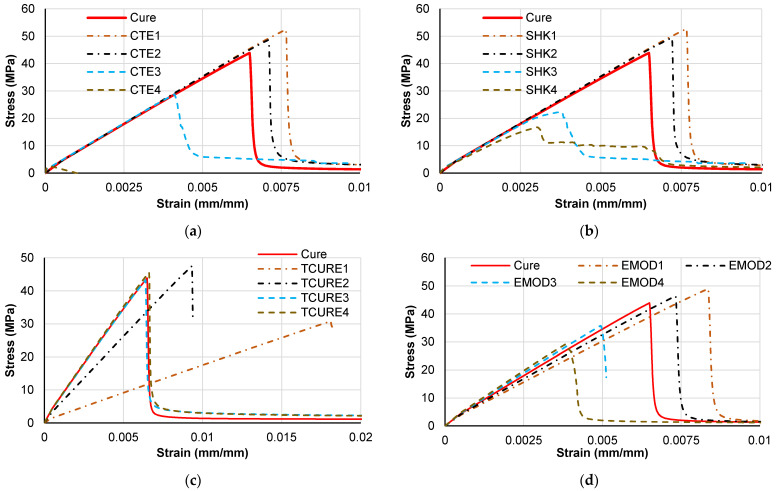
Tensile stress–strain response sensitivity to material and process parameters. (**a**) Thermal expansion sensitivity results, (**b**) Chemical shrinkage sensitivity results, (**c**) Cure temperature sensitivity results and (**d**) Elastic modulus sensitivity results.

Figure 17 shows the effective stress–strain relations, which present the expected tendency following the micro-residual stress analyses from Figure 16. The identification of the curves corresponds to Table 5; for example, CTE1 corresponds to the CTE value of case 1. The increase in micro-residual stresses generates a direct reduction of the material tensile strength.

The cure temperature reduction leads to a reduction in the elastic modulus followed by a loss of material strength (although resin strength parameters are kept fixed). The variation of the elastic modulus has a direct effect in the composite stiffness, as expected, and leads to a tensile strength reduction due to the higher micro-residual stress levels.

**Figure 18:**
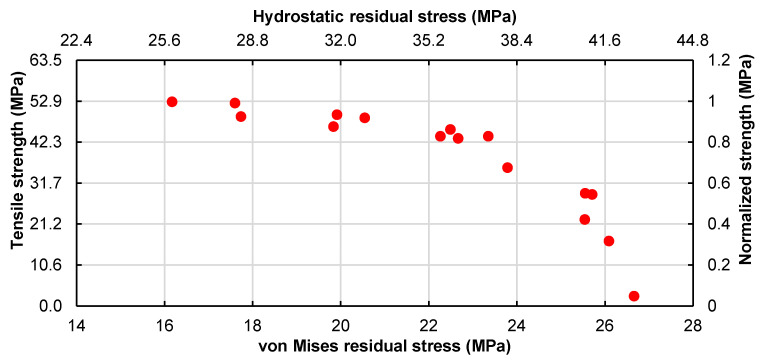
Impact of the average residual stresses on the strength reduction.

The sensitivity results show that the strength reduction can be mainly attributed to the micro-residual stress state that was characterized by the average von Mises stress and hydrostatic stress. Using all the data obtained above and plotting the tensile strength against the von Mises stress and the hydrostatic component, as shown in Figure 18, two results are retrieved. First, the von Mises stress follows a linear relation with the average hydrostatic component for the curing micro-residual stresses state. Second, independently of the parameter that is being modified, the micro-residual stress effects hold, allowing us to conclude, within the scope of the current work, that the loss of material performance (tensile strength) is a direct consequence of the micro-residual stress levels.

Although it is recognized that additional work is required to appropriately understand the influence of process parameters on the mechanical response of the matrix resin at the micromechanical level, the difficulties associated to experimental work at this scale make the numerical insight a valuable tool to understand better the effective macro-mechanical response of composite materials, as demonstrated by the methodology proposed in this work.

## 4. Conclusions

The micro-residual stress analysis showed that stress concentrations are retrieved between the fibers in the narrower gaps, while the higher hydrostatic components are present in the resin-rich regions. This effect is clearly attributed to the constraining effect that the fiber arrangement imposes on the matrix, because the whole RVE is in traction-free condition.

During the curing process, the whole RVE experiences volume changes that follow the superposition of the CTE effects during heating, chemical shrinkage during curing, and the final thermal contraction during cooling down. This superposition gives a final volume reduction for the current example of 0.9%, approximately half of the chemical shrinkage (2%).

The micro-residual stresses have almost no influence in the effective stiffness of the composite system, but they have a more considerable effect in the strength properties, for example, reducing the transverse tensile strength by 11%. However, the experimental measured strength properties with macro-scale coupons are still slightly lower than the predicted properties considering the curing conditions.

The sensitivity analysis shows that increasing the CTE, chemical shrinkage and elastic modulus contributes to an increase of the micro-residual stresses. The cure temperature influences the final degree of cure of the material, degrading the elastic modulus.

A direct analysis of the transverse tensile strength as a function of the micro-residual stresses shows a linear relation between the von Mises stress and the hydrostatic stress. Therefore, the loss of performance can be attributed to the micro-residual stresses.

## Figures and Tables

**Table 1:** Carbon fiber material properties.

Properties	Value
Fiber diameter (mm)	7 × 10^−3^
Density (kg/m^3^)	1800
Specific Heat (kJ/kg·K)	0.752
Thermal Conductivity (W/m·K)	9.38
$CTE_{L}$ (°C^−1^)	−0.38 × 10^−6^
$CTE_{T}$ (°C^−1^)	6.94 × 10^−6^
$E_{L}$ (GPa)	230
$E_{T}$ (GPa)	15
$v_{L}$ (n.d)	0.2
$G_{L}$ (GPa)	15
$G_{T}$ (GPa)	7

**Table 2:** Resin system material properties.

Properties	Value
Density (kg/m^3^)	1310
Specific Heat (kJ/kg·K)	0.679
Thermal Cond. (W/m·K)	0.15 ^1^
CTE (10^−6^ °C^−1^)	61.0 ^1^
Shrinkage (%)	2.0 ^1^
$T_{g}$ (°C)	135 ^2^
$E_{m}$ (MPa)	2850
$v_{m}$ (n.d)	0.33
$v_{p}$ (n.d)	0.30 ^1^
$S_{y+}$ (MPa)	55
$S_{y-}$ (MPa)	81 ^3^
$S_{u+}$ (MPa)	65
$S_{u-}$ (MPa)	93 ^3^
$\varepsilon_{f}$ (%)	2.6
$G_{flm}$ (N/mm)	0.12 ^3^

^1^ Estimated from literature for similar epoxies. ^2^ From the storage modulus onset. ^3^ Estimated from experimental tests with glass fiber prepregs. The compression values were extrapolated from transverse compression tests and the fracture energy corresponds to the interlaminar fracture toughness measured with DCB tests according to ASTM 5528.

**Table 3:** Loading cases to identify the effective composite properties.

Test	Description	Elastic Tensor Parameters	Yield Surface Parameters	Failure Surface Parameters
Longitudinal uniaxial tension	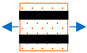	$E_{L}$; $v_{L}$	-	-
Transverse uniaxial tension ^1^	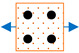	$E_{T};\;v_{T}$	$Y_{+UT}$	$S_{+UT}$
Transverse shear	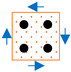	$G_{T}$	$Y_{ST}$	$S_{ST}$
Longitudinal shear	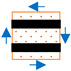	$G_{L}$	$Y_{SL}$	$S_{SL}$

^1^ Although $v_{T}$ is not required to identify the elastic tensor, it can be easily determined.

**Table 4:** Resin curing cycle.

**Time (min)**	0	23.25	38.25	50	110	130
**Temperature (°C)**	25	85	85	120	120	20

**Table 5:** Set of parameters used in the sensitivity analysis.

Parameter	Value				
	Nominal	Case 1	Case 2	Case 3	Case 4
Shrinkage (mm/mm)	−0.02	−0.01	−0.015	−0.025	−0.03
CTE (10^−6^ °C^−1^)	60	20	40	80	100
Elastic mod. (MPa)	2280	2565	2850	3135	3420
T cure (°C)	120	90	105	130	-

**Table 6:** Fitting parameters for the curing kinetics model.

$A_{1}\;(1/s)$	$\Delta E_{1}\;(\mathrm{kJ}/\mathrm{mol})$	$A_{2}\;(1/s)$	$\Delta E_{2}\;(\mathrm{kJ}/\mathrm{mol})$	n	m
3.24 × 10^15^	134,627	0	0	1.00	0

**Table 7:** Ply material properties measured from experimental tests.

Test Standard	Property	Average	Standard Deviation
ASTM D3039	$E_{T}$ (MPa)	7313	192
ASTM D3039	$v_{L}$ (n/d)	0.0109	0.0044
ASTM D5379	$G_{T}$ (MPa)	2812.8	73.9
ASTM D3518	$G_{L}$ (MPa)	3215	187
ASTM D3039	$S_{+UT}$ (MPa)	42.91	4.15
ASTM D5379	$S_{ST}$ (MPa)	22.34	2.17
ASTM D3518	$S_{SL}$ (MPa)	21.41	1.577

**Table 8:** Effective mechanical properties comparison.

	Experimental Results	Numerical Predictions		Experiments vs. Cure Predictions (%)
		Cure	No-Cure	
$E_{22}$ (MPa)	7313 ± 192	7151 ± 114	7235 ± 55	2.3
$v_{21}$ (n/d)	0.0109 ± 0.0044	0.0115 ± 0.0002	0.0115 ± 0.0002	−5.1
$v_{23}$ (n/d)	0.34 ^1^	0.34 ± 0.01	0.34 ± 0.01	−0.6
$G_{23}$ (MPa)	2729 ± 132	2674 ± 71	2733 ± 74	2.0
$G_{12}$ (MPa)	3215 ± 187	3353 ± 63	3460 ± 80	−4.3
$S_{+UT}$ (MPa)	42.9 ± 4.2	46.0 ± 2.0	51.9 ± 1.8	−7.3
$S_{ST}$ (MPa)	22.3 ± 2.2	25.0 ± 2.6	32.3 ± 0.7	−12.2
$S_{SL}$ (MPa)	21.4 ± 1.6	23.5 ± 1.2	26.8 ± 0.8	−9.6

^1^ Estimated from shear modulus.
